# Urinary bladder carcinoma with triplicate differentiations into giant cell sarcomatoid carcinoma, squamous cell carcinoma, and papillary urothelial transitional cell carcinoma: a case report

**DOI:** 10.1186/1757-1626-2-9111

**Published:** 2009-11-30

**Authors:** Tadashi Terada

**Affiliations:** 1 Department of Pathology, Shizuoka City Shimizu Hospital, Shizuoka, Miyakami 1231 Shimizu-Ku, Shizuoka 424-8636, Japan

## Abstract

The author reports a very rare and very unique urinary bladder carcinoma. This carcinoma occurred in a 68-year-old Japanese patient who underwent cystectomy for bladder tumor. The tumor was large polypoid and ulcerated one. Histologically, the tumor consisted of the following three elements: giant cell sarcomatoid carcinoma (70% in area), squamous cell carcinoma (20% in area), and papillary urothelial transitional cell carcinoma (10% in area). The former two elements were invasive into the peribladder fat tissue, while the latter one element invaded into the submucosa. There were gradual merges between the giant cell sarcomatoid element and squamous cell carcinoma element. Apparent transitions were not seen between the transitional cell carcinoma element and the giant cell sarcomatoid element or the squamous cell carcinoma element. Immunohistochemically, the giant cell sarcomatoid element was positive for various kinds of cytokeratins and vimentin while the squamous and transitional cell carcinoma elements were positive for various kinds of cytokeratins and negative for vimentin. The giant cell sarcomatoid element was free of other specific antigens. The author speculates that giant cell sarcomatoid carcinoma transdifferentiates into squamous cell carcinoma, and vice versa. The relationship between transitional cell carcinoma and the other two elements is unclear in the present case.

## Introduction

Sarcomatoid carcinoma is a well known entity of the urinary bladder [1-8]. Squamous cell carcinoma of the urinary is well known to occur in the urinary bladder [9]. In most of these cases, foci of urothelial carcinoma are present within such tumors. However, sarcomatoid carcinomas of the urinary bladder with compoments of squamous cell carcinoma and urothelial transitional cell carcinoma have not been reported, to the best of the author's knowledge. Here, the author reports a unique and very rare case of giant cell sarcomatoid carcinoma with squamous cell carcinoma component and papillary urothelial transitional cell carcinoma component.

## Case presentation

A 68-year-old Japanese man was admitted to our hospital because of sudden hematuria and abdominal pain. Endoscopic and imaging techniques including CT, MRI, and PET revealed a bladder tumor. Transurethral biopsy and clinical cytology revealed malignant tumor composed of giant cells, and cystectomy was performed. Macroscopically, the bladder contained a large polypoid and ulcerated reddish tan tumor (5 × 6 cm).

**Figure 1:**
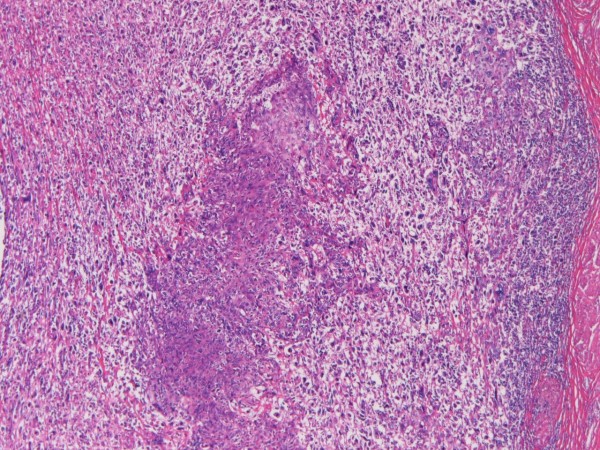
**The bladder tumor**. The tumor consists of squamous cell carcinoma (center) and giant cell sarcomatoid carcinoma. HE, ×40.

For histological evaluation, 17 tissue specimens were obtained from the tumor. Histologically, the tumor consisted of the following three elements: giant cell sarcomatoid carcinoma (70% in area), squamous cell carcinoma (20% in area), and papillary urothelial transitional cell carcinoma (10% in area) (Figure 1). The giant cell sarcomatous element and squamous cell carcinoma element were invasive into the peribladder fat tissue, while the transitional cell carcinoma element was invasive into the submucosa.

**Figure 2:**
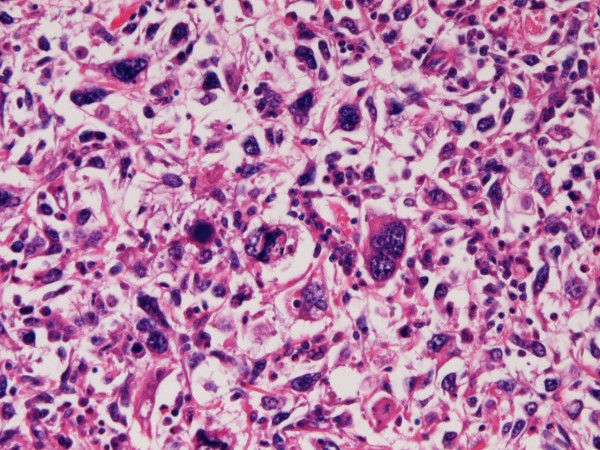
**High power view of sarcomatoid carcinoma**. It is composed of giant cells of spindle and polygonal shapes. The pleomorphism is very high. Many malignant multinucleated giant cells are also recognized. HE, ×200.

The sarcomatoid element was composed of malignant polygonal and spindle giant cells with frequent multinucleated giant cells (Figure 2). The giant cells had hyperchromatic nuclei and showed many mitotic figures. The giant cell sarcomatoid carcinoma area was homologous, and did not show heterologous elements such as bones and cartilages. Specific structures including herring bone and storiform appearances were not recognized. The tumor cells did not show plasmacytoid features. Lymphocytic infiltration was absent.

**Figure 3:**
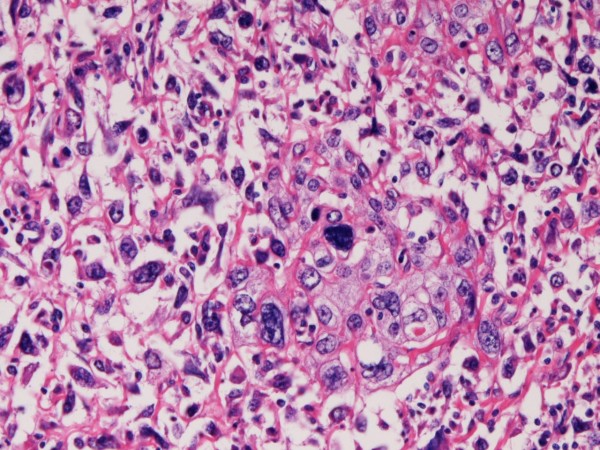
**High power view of a small focus of squamous cell carcinoma**. Transitions between squamous cell carcinoma and giant cell sarcomatoid carcinoma are recognized. HE, ×200.

The squamous cell carcinoma element was composed of malignant squamous cell with individual keratinization and intercellular bridge formation. Many mitotic figures were recognized in the squamous cell carcinoma element. There were gradual merges between the giant cell sarcomatoid and squamous cell carcinoma elements (Figure 3). There were occasional lymphovascular permeations by the giant cell sarcomatoid carcinoma and squamous cell carcinoma elements.

The papillary urothelial transitional cell carcinoma was grade II carcinoma with a few lymphovascular permeations. No transitions were observed between the transitional cell carcinoma element and the other two elements.

An immunohistochemical study was performed with the use of Dako Envision method, as previously reported [10,11]. The reagents and results are shown in Table 1. The giant cell sarcomatoid carcinoma element was positive for various kinds of cytokeratins, epithelial membrane antigen, and vimentin. It was negative for other mesenchymal markers such as desmin and S100 protein. P53 protein was positive and Ki-67 labeling was 72%. It was negative for CD68. The squamous cell carcinoma element was strongly positive for various types of cytokeratins and epithelial membrane, but was negative for vimentin and other mesenchymal markers. Neuroendocrine antigens and melanoma antigens were absent. P53 was positive, and Ki-67 labeling was 72%. The papillary urothelial transitional cell carcinoma element showed the similar results to those of the squamous cell carcinoma element, but the former was positive for cytokeratin 20 (Table 1). Both the epithelial elements were positive for p53 and showed high Ki67 labeling (squamous cell carcinoma, 67%; transitional cell carcinoma, 32%). Other antigens examined were negative (Table 1).

**Table 1:** Reagents and results in immunohistochemistry.

Antigens	Antibodies (clone)	Sources	Giant cell sarcomatoid	SCC	TCC
Pancytokeratin	AE1/3	Dako Corp. Glostrup, Denmark	++	+++	+++

Pancytokeratin	CAM5.2	Becton Dickinson Co. CA, USA	++	+++	+++

HMWCK	34βE12	Dako	+	+	++

CK5/6	D5/16	Dako	+	++	++

CK7	N1626	Dako	+	+	++

CK8	35βH11	Dako	+	++	++

CK14	LL002	Novocastra, Newcastle upon type, UK	+	+	++

CK 18	DC10	Dako	++	+++	+++

CK 19	RCK 108	Progen, Heidelberg, Germany	-	+	+

CK20	K20.8	Dako	-	-	++

EMA	E29	Dako	+	+	++

Chromogranin	DAK-A3	Dako	-	-	-

Synaptophysin	Polyconal	Dako	-	-	-

CD56	MOC-1	Dako	-	-	-

CD68	KP-1	Dako	-	-	-

Melanosome	HMB45	Dako	-	-	-

Vimentin	Vim 3B4	Dako	+++	-	-

Desmin	D33	Dako	-	-	-

S100 protein	polyclonal	Dako	-	-	-

ASMA	1A4	Dako	-	-	-

Myoglobin	polyclonal	Dako	-	-	-

CD34	NU-4A1	Nichirei, Tokyo, Japn	-	-	-

p53 protein	DO-7	Dako	++	++	+

Ki-67	MIB-I	Dako	72%	67%	32%

SCC, squamous cell carcinoma; TCC, transitional cell carcinoma area; +++, 67-100% positive; ++, 33-66%; +, 1-33% positive; -, negative; HMWCK, high molecular weight cytokerain; CK, cytokeratin; EMA, epithelial membrane antigen; ASMA, α-smooth muscle antigen.

A diagnosis of bladder carcinoma composed of three different elements was made. The patient was treated by palliative chemotherapy and radiation, but died of systemic metastases 32 months after the operation. Autopsy was not performed.

## Discussion

In the present case, the percentage of areas of each element was as follows: giant cell sarcomatoid carcinoma (70% in area), and squamous cell carcinoma (20% in area), and papillary transitional cell carcinoma (10% in area). The sarcomatoid element predominated over other elements. Therefore, the present cases can be labeled as giant cell sarcomatoid carcinoma.

The present case is not carcinocarcoma or sarcoma, because the sarcomatoid giant tumor cells were positive for cytokeratins and vimentin and negative for other mesenchymal antigens. The giant cell sarcomatoid component of the present case is different from "sarcomatous stroma", which is a bladder sarcomatoid reaction like nodular fasciitis of the skin [12,13]. It often occurs after bladder mucosal injuries. It is characterized by edema and inflammatory infiltration, and is usually negative for cytokeratins and positive for smooth muscle actin or vimentin [12,13]. The present case lacked such features of "sarcomatoid stroma" of the urinary bladder. The giant cells of the present giant cell sarcomatoid carcinoma are different from osteoclast-like giant cells rarely seen in sarcomatoid carcinoma [7,14], because the giant cells were positive for cytokeratins and negative for CD68 and also because the histologies of giant cells were entirely different from the osteoclast-like giant cells. The present sarcomatoid carcinoma is not rarely-reported giant cell tumor [15], because of different morphologies and because the present tumor expressed cytokeratins. The giant cell sarcomatoid carcinoma area in the present case showed homologous histology, and features of storiform-pleomorphic, herring bone, formations of bones and cartilages, plasmacytoid, and lymphoepithelial carcinoma are absent. The area was negative for neuroendocrine antigens, so that the area is not large cell neuroendocrine carcinoma. The present tumor is not malignant melanoma.

The pathogenesis of sarcomatoid carcinoma of the urinary bladder is only speculative despite of many studies [1-8]. Almost all cases of sarcomatoid carcinoma of the bladder contain foci of urothelial transitional cells carcinoma [1-8]. Previous studies have suggested that the sarcomatous component of sarcomatoid bladder carcinoma is derived from urothelial carcinoma. Interestingly, Sung et al. [8] have recently suggested the monoclonal nature of sarcomatoid bladder carcinoma with the use of molecular techniques. This finding strengthens the concept that the bladder sarcomatoid carcinoma is monoclonal lesion derived from urothelial carcinoma.

In the present study, there were close associations between giant cell sarcomatoid carcinoma element and squamous cell carcinoma element. There were frequent histological merges between the two elements. This may indicate that the squamous cell carcinoma element in the present case was derived from trans-differentiation from the giant cell sarcomatoid carcinoma element, or that the giant cell sarcomatoid carcinoma element was derived from the squamous cell carcinoma element. The present study strongly suggested the monoclonal natures of the sarcomatoid carcinoma and squamous cell carcinoma in bladder sarcomatoid carcinomas. Much more studies using molecular genetic analyses such as LOH analyses are required in sarcomatoid carcinomas of the bladder.

The present study did not show spatial relationship between urothelial papillary transitional cell carcinoma and the other two elements. Previous studies have suggested that the sarcomatoid area of the bladder sarcomatoid carcinoma is derived from urothelial transitional carcinoma [1-8]. It is possible that in the present study, sarcomatoid carcinoma and squamous cell carcinoma elements are not associated with urothelial transitional cell carcinoma element. However, this is not always true. Much more studies using a large series and molecular analyses of bladder sarcomatoid carcinoma is mandatory to determine the pathogenesis of this rare tumor.

Finally, sarcomatoid carcinoma of the urinary bladder tends to be high grade and shows a poor prognosis than usual urothelial carcinoma [1-8]. In the present case, the survival period was 32 months after the operation. A critical studies on the mechanism(s) of the development of sarcomatoid carcinoma of the urinary bladder are needed to obtain the radical therapy for this rare neoplasm.

## Consent

Written informed consent was obtained from the patient for publication of this case report and accompanying images. A copy of the written consent is available for review by the Editor-in-Chief of this journal.

## Competing interests

The author declares that they have no competing interests.
